# Give Them a Toy or Increase Time out of Kennel at Lawn Areas: What Is the Influence of These Interventions on Police Dogs’ Welfare?

**DOI:** 10.3390/ani11082264

**Published:** 2021-07-30

**Authors:** Letícia Bicudo Nogueira, Rupert Palme, Olívia Mendonça-Furtado

**Affiliations:** 1 Faculdade de Medicina Veterinária e Zootecnia, Universidade de São Paulo, São Paulo, SP 05508-270, Brazil; 2 Unit of Physiology, Pathophysiology, and Experimental Endocrinology, Department of Biomedical Sciences, University of Veterinary Medicine Vienna, 1210 Vienna, Austria; Rupert.Palme@vetmeduni.ac.at; 3 Departamento de Psicologia Experimental, Instituto de Psicologia, Universidade de São Paulo, São Paulo, SP 05508-030, Brazil; mendoncafp@alumni.usp.br; 4 National Institute for the Atlantic Forest, Santa Teresa, ES 29650-000, Brazil

**Keywords:** kennelling, confinement stress, stereotypies, faecal cortisol metabolites, police dogs, working dogs, environmental enrichment, animal welfare

## Abstract

**Simple Summary:**

Poor environments such as kennels can lead to compromised welfare, as they usually lack many kinds of stimuli. Working dogs are not only kept in kennels, but they are also often housed without any kind of environmental enrichment, allegedly due to motivational reasons, which is conducive to all kinds of behavioural issues. Thus, this study aimed to evaluate the impact of two interventions, a toy and going out to a lawn area, on a group of police dogs. Behavioural and physiological data were used to evaluate the animals’ responses to treatments. We identified a large variety of behavioural profiles amongst the animals that presented different levels of stereotypies, which is an abnormal behaviour pattern that can be associated with stress. A tendency to a reduction in these behaviours in high-stereotyping individuals was observed after dogs were able to go to the lawn area, indicating beneficial effects of this intervention. The physiological data indicated that the cumulative effect of interventions can also have a beneficial impact and that environmental enrichment plays an important role in kennelled police dogs’ welfare.

**Abstract:**

This work was aimed at identifying the effectiveness of two interventions applied to a group of eight kennelled police dogs. Interventions consisted of access to a lawn area (350 m^2^) and “toy” (a 30 cm jute rag roll, hanging from the kennel ceiling), both available for 15 min a day, for four days in a row. We collected behavioural data and faecal samples for cortisol metabolites evaluation before, during and after interventions. Faecal cortisol metabolites levels were significantly reduced (Friedman, X2(3) = 12.450; *p* = 0.006) during the second round of intervention, regardless of the type of intervention, indicating that the interventions can have a beneficial cumulative effect. Regarding the frequency of stereotyped behaviour, cluster analysis identified two groups of individuals: (1) high-stereotyping individuals (n = 3) that had a tendency to reduce stereotyping behaviours in the lawn intervention when compared to toy intervention (Friedman, X2(3) = 2.530; *p* = 0.068), and (2) low-stereotyping animals (n = 5) that did not present significant behavioural changes during the experiment. The variety of behavioural and endocrine parameters evaluated highlights the need to account for the individual in behaviour and welfare research. Overall, our results suggest that even simple environmental enrichment can be an effective method to mitigate behaviour and physiological signs of stress.

## 1. Introduction

The existence of an association between the development of stereotyped behaviours and sub-optimal environments is widely recognized [1,2,3]. In kennelled domestic dogs, this link has been described by several authors, who interpreted repetitive behaviours as indicative of compromised welfare [4,5,6,7,8] and chronic stress [9,10,11,12,13,14,15,16]. Kennelled dogs may experience poor housing as this management can be associated with lack of control of the environment [17], unpredictability [12,18] and limited opportunities for social contact with humans [5,19] and/or other dogs [6] (for a definition and the importance of welfare for kennel dogs, see [13]). Thus, many efforts to investigate how to provide adequate stimulation in this situation have been made, and several studies have indicated that environmental enrichment is effective in minimizing kennelling stress [4,18,20,21,22,23,24].

Military/police working dogs are commonly housed in kennels (Belgium [8,25]; Brazil [26,27]; France [28]; The United States [29]; The United Kingdom [13,22]), and many of these dogs do not receive any kind of environmental enrichment, due to a widespread belief that providing some types of stimuli would decrease animals’ motivation to work because they are trained using toys as rewards [22]. However, current evidence indicates that environmental enrichment does not impair a dog’s working performance [22] and also has a stress reducing potential [8]. For an environmental enrichment program to be successful, it requires considering both the motivation of the subjects to interact with the provided stimuli [30] as well as how individuals can differ in the way they deal with environmental challenges [31]. Thus, it is important to investigate the best way of providing effective stimuli to target subjects.

Previous research on long-term kennelled dogs has focused on laboratory [5,21] and shelter dogs [20,32]. Toys, training routines and infrastructural environmental enrichment were demonstrated to be effective ways to enhance the welfare of those animals, by increasing exploration, reducing abnormal behaviours, promoting interaction with enrichment, increasing activity and lowering plasma cortisol levels. In working dogs, Lefebvre et al. [8] have compared two regimes of guided exercise practicing and training sessions based on a welfare perspective. They found reduced cortisol concentrations when the enrichment was more frequent. Nevertheless, further work is required to evaluate the effect of different kinds of interventions on the welfare of working dogs.

In an effort to contribute to the working dog literature, we evaluate the effectiveness of two different stimuli (access to a lawn area and “toy”) provided to a group of kennelled police dogs; we measured behavioural and physiological parameters before, during and after the interventions. Although we expected both interventions to be beneficial to the dog’s welfare, in response to the increase of stimuli, we hypothesized that the lawn intervention would be more efficient in reducing stress, leading to a reduction in repetitive behaviours and faecal cortisol metabolite levels because dogs would be able to reduce time spent inside kennels and have access to a great variety of stimuli, allowing better control of the environment.

## 2. Materials and Methods

### 2.1. Subjetcs

Subjects comprised eight police dogs from the city Guard of Santana do Parnaíba—SP, Brazil (Table 1). Most of the dogs had been born and raised in working dog training establishments, except subject No. 4, which came from a civilian house as a young adult.

### 2.2. Husbandry and Housing

Activities practiced by the dogs included scent detection and guard function. Dogs’ routine was based on a scale of 12 h of “work”, when they could be trained and take part in the city patrol, per 36 h of “rest”, when they were kept exclusively inside the kennels until their next workday. Each dog was designated to a guard that was both their handler and the caretaker, so the work scales of the animals coincided with the ones from their respective handlers. This division was the same used for experimental groups (see Table 1) to guarantee that animals from the same group were submitted to the same events during duty. There were no care assistants; all the management procedures were realized by the guards. Dogs were fed twice a day, at 11:00 and 23:00. Kennels were cleaned at 11:30.

Housing conditions consisted of individual 11.5 m^2^ sized kennels with an internal subdivision of 2.3 m^2^ as a resting area. Three walls and the floor were made of concrete and the front wall was made of wire mesh. Walls were 2.5 m high, surrounding the entire structure. Sixty percent of the structure was covered by a roof. Visual contact was possible only with the animals housed on the kennel box across the aisle.

### 2.3. Interventions

Animals received two kinds of interventions: access to a lawn area and a “toy”. Lawn intervention consisted of the subject being allowed to move freely in a 300 m^2^ lawn area, with natural grass, surrounded by a fence. There was no social interaction as subjects were individually released in the area. The “toy” intervention consisted of providing a jute rag roll (the same material used in the guard training sessions), 50 cm × 20 cm, tied from both sides by a rope and hanging from the kennel ceiling about 1.2 m above the ground. As the dogs were not used to be handled by unfamiliar persons, guards were responsible for taking the subjects to the lawn area and tying the “toys” in the kennels spaces; however, they were advised not to interact with the subjects at any moment. Both interventions had a fifteen-minute duration.

### 2.4. Experimental Design

**Figure 1:**
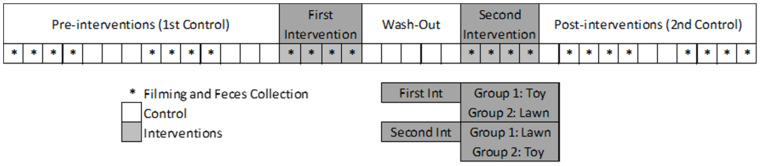
Experimental design utilized. Each square represents a day.

Interventions were applied during the “rest” period of the dog’s schedule, between 12:00 and 12:30, just after the kennels were cleaned. As the experimental groups were predefined by the duty scale, we could only determine the order each group would receive the interventions. The whole experiment took 38 days from January to February 2018. It had three parts: pre-, during- and post-interventions, with eight days of behavioural and physiological (faecal cortisol metabolites) measurements during each part (Figure 1), always at the same time of the day (see items 2.5 and 2.6 for more information). The study followed a crossover experimental design. Animals had access to each intervention during a continuous period of four days, separated by an interval of five days (wash-out period).

### 2.5. Behaviour Assessment

To minimize human-induced perturbations [8,33], animals were filmed without the researcher’s presence. Records of 30 consecutive minutes were made for each dog between 12:30 and 14:30, a time during which, by routine, all dogs were inside their kennels. Filming happened on the same days as the faeces collection (see next section). On the first day, the order that individuals were filmed were drew. Throughout the experiment, the same order was used, but the last animal on the previous day was always the first on the next day. A total of 96 h of video recordings was taken, 12 h for each subject (4 pre, 4 during and 4 post interventions).

Video recordings were used for behavioural data collection by a one-minute interval instantaneous sampling method [34]. The first two minutes of each video were removed from analysis to minimize the interference of human presence when positioning the cameras [35]. Therefore, each dog had a total of 672 scans registered. Behaviours were classified according to the ethogram presented in Table 2. Analyses were based on two sets of variables. The first one consisted of the behavioural categories: “Readiness/Alert”, “Stereotyped” and “Rest/Relaxation” (Table 2). The second one consisted of the specific behaviours: “Pace”, “Circle”, “Spin”, “Anxious waiting” and “Stand” as variables (Table 2). L.B.N. was the only one to collect behavioural data from the videos.

### 2.6. Faecal Samples

Faecal samples were collected from 11:30 to 12:00, during the cleaning of the kennels, totalling 85 samples. The collection could also happen after filming, when the animal had not defecated before the time of faecal collection or had done it long before (noticeable too dry faeces), to guarantee the freshest samples possible. On rare occasions, when the animal did not defecate on that day, we could not collect a sample. The whole stool was collected in a plastic bag, manually homogenized by kneading the bag, and then a small amount was transferred to a 5 mL identified plastic tube. Tubes were immediately stored in a cooler with ice bags and transported to the Laboratory of Behavioural Endocrinology at the Institute of Psychology of The University of São Paulo, where they were kept in a freezer until extraction and analysis of faecal cortisol metabolites (FCMs).

### 2.7. FCM Extraction and Analysis

The extraction procedure used 80% methanol (0.5 g faeces plus 5 mL of 80% methanol) following the protocol described in Palme [36]. The supernatant was stored at −20 °C until assayed. Faecal hormone metabolites were measured in a 50 µL aliquot of the extract (diluted 1:10) with a cortisol enzyme immunoassay (EIA; for details, see Palme and Möstl [37]), validated for *Canis familiaris* by Schatz and Palme [38]. Both intra- and inter-assay coefficients of variation of pool samples were <12%. All samples were assayed in duplicate. Concentrations of FCMs are expressed as nanograms per gram of wet faecal matter.

### 2.8. Ethics and Data Collection Procedures

This study was approved by the Animal Research Ethics Committee of the Institute of Psychology from The University of São Paulo (CEUA/IPUSP nº 1396090518). All the procedures were conducted in accordance with the ethical guidelines laid down by the National Council for Control of Animal Experimentation (CONCEA) and with the current Brazilian laws on ethical standards.

### 2.9. Data Analysis

When analysing the data, we noticed that subjects performed stereotyped behaviours in very distinct frequencies (see Results). Therefore, we ran a Hierarchical Cluster Analysis with the Centroid Clustering method, by the Squared Euclidean distance, using the mean values, per individual, of all stereotyped behaviours performed in the pre-intervention period (see Figure 1). The clusters classified individuals regarding the number of stereotyped behaviours they presented.

To test if there were differences between treatments (pre-interventions, lawn area, toy and post-interventions) and periods (pre-interventions, first intervention, second intervention and post-interventions) for both behaviours (categories: “Readiness/Alert”, “Stereotyped” and “Rest/Relaxation” and specific behaviours: “Pace”, “Circle”, “Spin”, “Anxious waiting” and “Stand”) and FCM concentrations, we used Friedman’s test.

Spearman’s correlation test was used to verify the existence of a correlation between (1) FCM level and behaviour variables (using the same categories and specific behaviours used in the previous analysis) and (2) the number of different behaviours performed by the individual (behavioural repertory) and the percentage of stereotyped behaviours performed.

We used non-parametric tests because the small sample size of our study (*n* = 8) precluded verifying normality distribution of data in all experimental situations (all data checked employing Shapiro–Wilk tests). All analysis was made using SPSS (IBM SPSS Statistics 22). Statistical significance was determined at a *p*-value of less than 0.05. The effects of breed, sex or age have not been tested.

## 3. Results

Stereotyped behaviour accounted for 16.7% of all scans in our sample. All dogs exhibited some type of stereotyped behaviour, but rates varied considerably among individuals (from 0.2 to 60.0%).

### 3.1. Cluster Analysis of Stereotyped Behaviours

**Figure 2:**
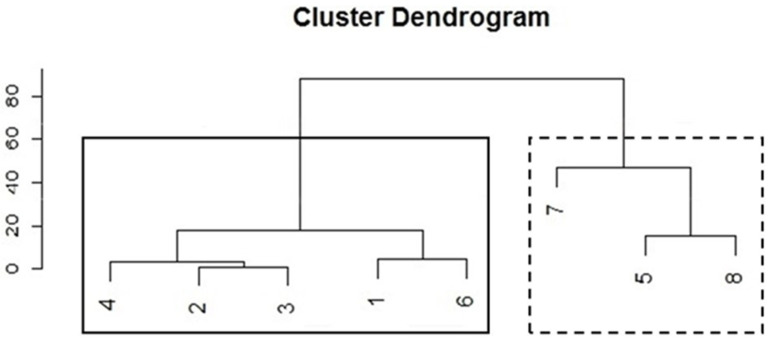
Cluster dendrogram ran with the percentage of observations of all stereotyped behaviours performed on pre-interventions period. Numbers indicate subjects (Table 1), the dotted line cluster indicates high-stereotyping individuals and the solid line cluster indicates low-stereotyping individuals.

**Figure A1:**
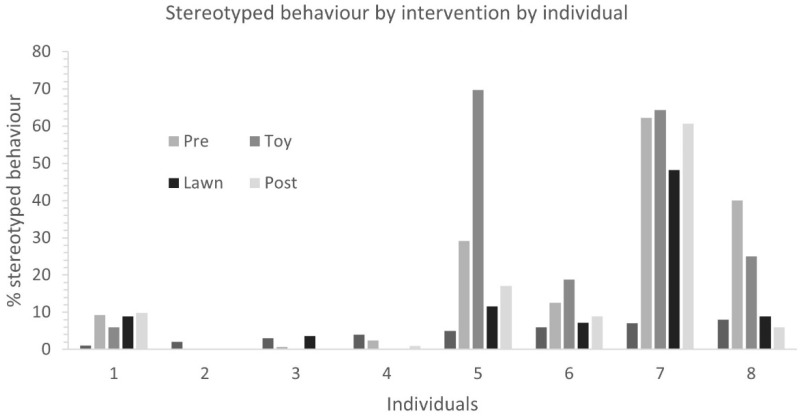
Percentage of observations on stereotyped behaviour by individual (numbers indicated on Table 1) in our four experimental treatments (Pre: pre-interventions; Toy: toy intervention; Lawn: lawn intervention; Post: post-interventions).

The cluster analysis recognized two patterns of behavioural responses (Figure 2): (1) subjects that displayed higher rates of stereotyped behaviours (*n* = 3, dotted line cluster), high-stereotyping individuals, and (2) subjects that displayed lower rates of stereotyped behaviours (*n* = 5, solid line cluster), low-stereotyping individuals. (See Figure A1).

### 3.2. Behavioural Analysis

#### 3.2.1. Readiness/Alert Behaviours

Readiness/Alert behaviours did not differ significantly between the four experimental situations (pre-intervention, both interventions and post-intervention), neither regarding periods (Friedman, X^2^ (3) = 1.950; *p* > 0.05) nor regarding treatments (Friedman, X^2^ (3) = 2.250; *p* > 0.05). Even when considering only high-stereotyping individuals, there were no statistical differences (Friedman, X^2^ (3) = 1.000; *p* > 0.05).

#### 3.2.2. Stereotyped Behaviours

**Figure 3:**
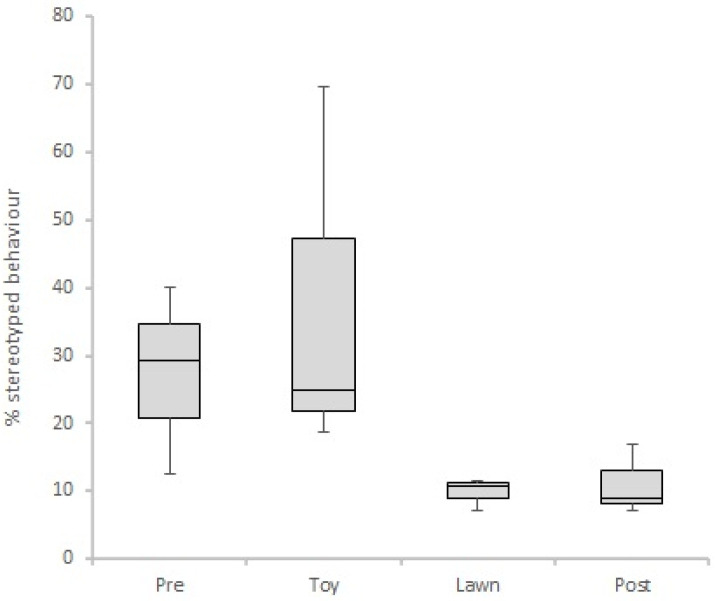
Boxplot of percentage of stereotyped behaviour in our four experimental treatments (Pre: pre-interventions; Toy: toy intervention; Lawn: lawn intervention; Post: post-interventions). Analysis of only high-stereotyping individuals.

Analysis, ran with the entire sample, did not present significant differences in stereotyped behaviours frequency, neither between periods (Friedman, X^2^ (3) = 4.897; *p* > 0.05), nor between treatments (Friedman, X^2^ (3) = 5.426; *p* > 0.05). The analysis made with only animals from high-stereotyping cluster showed a significant difference between treatments (Friedman, X^2^ (3) = 8.200; *p* = 0.042). However, when checking the pairwise comparison (Table 3) with Bonferroni correction, there was no *p*-value below 0.05, which suggests that the small sample of high-stereotyping individuals hinders any significant statistical result. Nevertheless, even with the Bonferroni correction, we observed a tendency that during the lawn treatment there was less engagement on stereotyped behaviours when compared to the toy intervention treatment (Friedman, X^2^ (3) = 2.530; *p* = 0.068) (Figure 3). Analysis by period found no statistical differences over time (Friedman, X^2^ (3) = 3.400; *p* > 0. 05).

#### 3.2.3. Rest/Relaxation Behaviours

Rest/Relaxation behaviours did not differ significantly between the four experimental situations, neither in analysis by treatments (Friedman, X^2^ (3) = 4.050; *p* > 0.05) nor in analysis by period (Friedman, X^2^ (3) = 1.050; *p* > 0.05). When the test was performed only with the animals grouped in the high-stereotyping cluster, statistical differences were found between treatments (Friedman, X^2^ (3) = 8.200; *p* = 0.042). When checking the pairwise comparison with Bonferroni correction, again, there was no *p*-value below 0.05. Nevertheless, even with the adjusted significance, we could see a tendency that the percentage of rest/relaxation behaviours was higher in the period animals had access to the lawn area compared to the period when animals received the toy (Table 4). Analysis by period found no statistical differences over time (Friedman, X^2^ (3) = 3.400; *p* > 0.05).

#### 3.2.4. Analysis of Specific Behaviours

Statistical analysis of specific stereotyped behaviours (Pace, Circle and Spin) by both treatment and period found no significant difference. The behaviours “Wall-bounce” and “Head twirl” could not be analysed due to low percentages of observations. The behaviours “Anxious waiting” and “Stand”, which were categorized as Readiness/Alert behaviour, were analysed as possible stress indicators, but showed no significant differences, neither by treatment nor by period.

### 3.3. Behavioural Repertory

**Figure 4:**
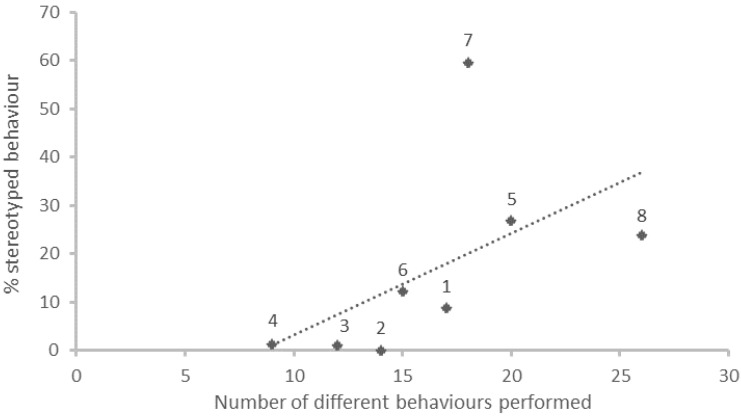
Graph presenting the linear correlation coefficient between the percentage of stereotyped behaviours presented by each individual, and the extension of their respective behavioural repertories (number of different behaviours performed). Individual values are represented by the diamonds.

The number of different behaviours performed by each animal was positively correlated (Spearman, ρ = 0.786; *p* < 0.05) with the percentage of stereotyped behaviours (Figure 4), and negatively correlated (Spearman, ρ = −0.810; *p* < 0.05) with the percentage of rest/relaxation behaviours performed by the same subject.

### 3.4. Correlations between Behaviour and FCM Levels

There were no correlations between behavioural categories (Readiness/Alert, r = 0.262; Stereotyped, r = 0.164; Rest/Relaxation, r = −0.235) or specific behaviours (r = −0.235; Pace, r = 0.370; Spin, r = 0.034; Circle, r = −0.308; Anxious Waiting, r = −0.053; Stand, r = 0.087) and FCM levels.

### 3.5. FCM Levels Analysis

**Figure 5:**
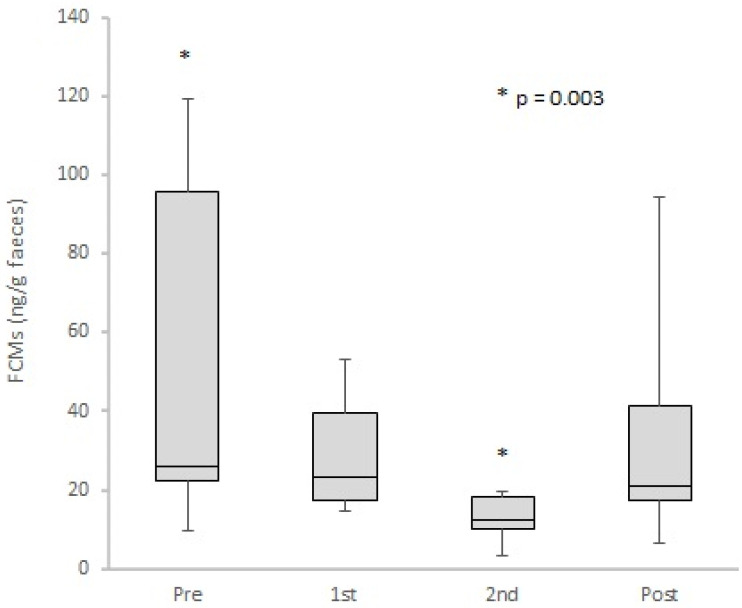
Boxplot of faecal cortisol metabolite (FCM) concentrations of all individuals in the four experimental periods (Pre: pre-interventions; 1st: first intervention; 2nd: second intervention; Post: post-interventions). Asterisks indicate significant differences between the two periods: Pre and 2nd.

FCM concentrations differed significantly between periods (Friedman, X^2^ (3) = 12.450; *p* = 0.006); the second period of interventions presented lower FCM concentrations than the pre-interventions period (Friedman, X^2^ (3) = 3.486; *p* = 0.003) (Figure 5). Graph with data on FCM throughout experimental periods by individual can be found on Supplementary Material (Figure S1). No statistical differences were found between treatments (Friedman, X^2^ (3) = 6.300; *p* > 0.05).

## 4. Discussion

Our subjects had higher (inter-subject mean = 16.7%) proportions of stereotyped behaviours on the behavioural budget than what has been reported in the literature for individually kennelled dogs (between <0.1% and 11% [4,39,40,41]). This could be explained by the fact that our subjects were working dogs with a very rigid management policy. Additionally, six out of our eight subjects were Malinois Shepherds, a breed bred to work as police dogs that shows a high correlation between performance in the field and stereotyped behaviour [42]. Furthermore, Malinois Shepherds were reported to have higher rates of stereotyped behaviours (4.8 to 10.6%, Lefebvre et al. [8], and 29.78 to 33.86%, Haverbeke et al. [25]) than other breeds.

It is noteworthy that, even in the absence of arousing stimuli, all our subjects presented some kind of stereotyped behaviour, which contrast sharply with available data for individually kennelled dogs performing stereotyped behaviours (1.3% to 15% [4,11,39,40]). Similar rates (93%) of subjects presenting stereotyped behaviour were described by Denham et al. [14], but only when their subjects were presented with arousing stimuli (e.g., care assistants walking through the kennel, the sound of clicking the clip on the end of a leash, sounds of feeding preparation, stranger standing outside kennel).

The cluster analysis pointed towards two different patterns of stereotype performance; three individuals were classified as high-stereotyping and five as low-stereotyping. Other studies on kennelled dogs also found individual differences in animals experiencing the same housing and management conditions [7,11,14,15,39,43,44,45,46,47,48,49]. Some authors propose that these variations are due to the existence of distinct personalities [50] or coping styles [51]. Stereotypies are rated as a proactive response to stress, whilst depression/inactivity is related to reactive’ subjects [31]. Further studies could test our subjects’ personalities in order to check this hypothesis.

High-stereotyping individuals showed a trend toward reducing stereotype levels when given the opportunity to use a lawn area (versus when allowed to play with a toy), which is compatible with the work of Lefebvre et al. [8], which found a reduction of stereotyped behaviour (“repetitive gait”) in working dogs that had access to regular outdoor physical activities.

Additionally, in the high-stereotyping cluster there was a trend to increase Rest/Relaxation behaviours during the lawn intervention (versus when allowed to play with a toy). When taken together, these results show that during the lawn intervention period, animals ceased to perform stereotyped behaviour and engaged in Rest/Relaxation behaviours, suggesting a successful improvement of their welfare state [2,52]. Some theories relate stereotypies origin in carnivores as thwart attempts to display behaviours they cannot perform (e.g., mate, roam, hunt, or interact with conspecifics) in the captive environment [53]. Aligned with those theories, we hypothesize that our subjects showed a reduction of stereotypies due to the given possibility to display highly motivational behaviours (e.g., roam, dig, sniff), even if only for a short period of time.

In the same line, we suppose that the lack of influence of the toy intervention on stereotyped behaviours was because it did not promote the performance of highly motivated behaviours [54,55]. Thus, although the animals were observed interacting intensively with the toy (e.g., chasing, jumping and biting), it did not promote time out of the kennel confinement, possibly the most relevant aspect of the lawn intervention.

However, our data not only indicate that stereotypies were not reduced during the toy intervention, but they also suggest that they had a slight increase. It is already recognized that situations that are positive to welfare can contribute to elicit or maintain stereotypies. This could occur because the stereotype no longer indicates a frustration from a non-executed behaviour or a need to use repetition as a “calming effect” but has turned into a “habit” [56]. An increase in stereotypies has also been reported in mink in response to providing them “playballs” [57] and in arctic foxes in response to an increase in cage size [58]. Thus, although stereotypies are an important sign to detect potential suffering associated with sub-optimal environments, they never should be taken as an exclusive indicator of welfare, and research is still increasing our knowledge on how they develop and differ between species and individuals [56]. When it comes to domestic dogs, considering that our subjects performed intense physical activity when interacting with the toy, it is also important to consider that additional exercise was already described to increase “active behaviours” [43,59], which could lead to a repetitive movement in restrained spaces. Furthermore, in the work of Protopopova et al. [48], exercising did not prevent the expression of abnormal in-kennel behaviours. Therefore, possible approaches to comprehend this effect in our subjects could be related to the arousal and excitement brought by interacting with the toy, not necessarily associated with a decrease in the welfare state.

Regarding the five low-stereotyping subjects, stereotyped behaviour was not a good proxy for stress response. Therefore, we would expect them to show alteration in other behavioural frequencies (e.g., decreased frequencies of Rest/Relaxation behaviours after the interventions). However, the environment that these dogs are maintained in is so sterile that an animal that did present stereotypies did not have anything else to do besides resting.

Regarding the analysis of specific behaviours, they did not present a correlation with any of the treatments/periods. Unfortunately, our small sample hinders any conclusion in this regard.

Our analysis shows that subjects that presented more stereotypies also had a wider behaviour repertory than subjects with fewer stereotyped behaviours. Although it sounds counter intuitive to present more repetitive behaviours and also a higher variability of behaviours, a closer analysis shows that high-stereotyping subjects were more active than low-stereotyping ones. This result also highlights that stereotypies are not always connected to compromise welfare [56] and that low-stereotyping subjects are not necessarily in a better welfare condition. Evidence in this regard is that we found a correlation between lower levels of stereotypies and less complex behaviour repertory. Several authors reported that, in kennel confined domestic dogs, a possible response to chronic stress was a reduction of the behavioural repertory [14,21,33,47]. Additionally, according to Ijichi et al. [31], subjects presenting less complex behavioural repertoires would be dealing with a reduced perception in the excitatory stimuli [60], reducing feedback in the pathways linked with motivation and appetite behaviours [31], leading to depressive like behaviours.

We found no correlations between behaviour (evaluated in the categories Readiness/Alert, Stereotyped, Rest/Relaxation and isolated: Pace, Spin, Circle, Anxious Waiting, Stand) and FCM levels, a non-invasive measure of adrenocortical activity [61]. This is not completely unexpected because different studies on dogs have also reported the absence of a relationship between cortisol levels and behaviours [44,62]. Our findings also corroborate studies that evaluated the relationship between personality traits and cortisol levels in domestic dogs [63] and confined domestic cats [64,65,66].

FCM levels were lower during the second period of interventions, but not associated with one specific type of intervention. Lefebvre et al. [8] found a reduction of cortisol levels in working dogs in response to the establishment of regular dog-handler interactions. Therefore, this result could be due to the increase of human contact with the animals throughout the experiment (put on the leash to walk the animal to the lawn area or enter the kennel to hang the toy and take it off). A non-mutually excluding explanation would be that the combination of both interventions was necessary to result in reduced cortisol levels. Additionally, new stimuli can have a “challenge” effect on dogs [9,25]. Therefore, the routine alteration due to the start of the experiment could have challenged the coping mechanism of the subjects, hampering the effects of the first period of intervention on FCM levels. In addition, it is important to consider that the lower levels of FCM seen only in the second period could also indicate that animals were less aroused by the interventions throughout time.

## 5. Conclusions

Considering that high-stereotyping individuals showed a tendency towards a reduction in stereotyped behaviours, and that the second round of interventions was related to a reduction in FCMs, we assume that these indexes were proxies of impaired welfare related to kennel confinement.

Fifteen minutes of access to the lawn area showed an impact on stereotyped behaviour levels on high-stereotyping dogs. Although this does not necessarily mean a reduction in stress levels, it implies an improvement in welfare once stereotyping behaviours can be deleterious. Additionally, FCM data indicate that the cumulative effect of interventions might benefit all subjects. Further research could evaluate if the length and the type of the interventions are crucial for their effect on the animals’ welfare.

The literature clearly points out that working dogs are extremely affected by the confinement they are subject to. Our research shows the positive effect that simple interventions can have on those animals’ welfare. Therefore, veterinarians and other staff responsible for these animals should consider applying regular interventions in order to maximize their welfare.

## Figures and Tables

**Table 1:** Subjects of the study identified by breed, sex, age when arrived at Guard kennel and at the beginning of the experiment and the guard duty scale which they belonged to. Animals from the same scales worked together and had the “rest” period at the same time.

Subject	Breed *	Sex	Age When Arrived Guard Kennel	Age at Beginning of Experiment	Duty Scale
1	MAL	F	3 months	4 years 7 months	1
2	GS	M	1 year 6 months	1 year 8 months	2
3	MAL	F	3 months	4 years	1
4	RO	M	2 years 2 months	2 years 7 months	2
5	MAL	M	3 months	2 years 7 months	1
6	MAL	M	3 months	4 years 7 months	2
7	MAL	M	3 months	4 years 3 months	2
8	MAL	M	2 months	3 years 2 months	1

* MAL: Belgian Shepherd Malinois; GS: German Shepherd; RO: Rottweiler.

**Table 2:** Ethogram adapted from Hubrecht (1993) with behaviours and descriptions, organized into behavioural categories.

Category	Behaviour	Definition
Readiness/Alert	Anxious waiting	In front of the gate, eyes seeking, upright posture
	Stand	Standing on four legs.
	Walk	Ambulatory gait.
Stereotyped	Circle	Circular trajectory. At least three consecutive times.
	Pace	Walking back and forth along a boundary line, with no identified purpose (such as defecating, going to the water bowl, or reaching gate when listening to a noise).
	Spin	Quickly turning in a tight circle, pivoting about hind legs. At least three consecutive times.
	Wall-bounce	Jumping at the wall and rebounding off it.
	Head twirl	Circular movement of the head, when the dog is at the end of a route, before changing direction.
Rest/ Relaxation	Rest with head up	Lying down with eyes open or closed, head up.
	Rest with head down	Lying down with eyes open or closed, head down.
	Not seen	Dog laid down on a wooden bed in the resting area.
Others	Sit	Sitting on hind legs.
	Urinate	Urinating in a squatting position or with one leg cocked.
	Defecate	Squatting and defecating.
	Sniff ground	Directing nose to the ground.
	Dig	Scratching the floor with the forepaws.
	Climb	Climbing the kennel gate.
	Gnaw	Gnawing gate bars or other non-nutritive material.
	Autogroom	Licking, pulling out body hair.
	Scratch	Scratching body with one of the hind legs.
	Drink	Drinking/with the mouth at the water bowl.
	Run	Running gait.
	Aggressive barking	Loud, rough vocalization.
	Play with water	Playing at the water bowl using the forelegs or nose.
	Lick	Licking the floor or the walls.
	Interaction with different objects	Interacting with novel objects at the kennel, such as insects or leaves.

**Table 3:** Pairwise comparison of Friedman test ran with animals from the high-stereotyping cluster for the frequency of stereotyped behaviours in the four experimental treatments. Adjusted significance (Adj. Sig.): significance after Bonferroni correction was applied.

Sample 1/Sample 2	Test Statistic	Std. Error	Std. Test Statistic	Sig.	Adj. Sig.
Lawn/Post	1.000	1.054	0.949	0.343	1.000
Lawn/Pre	2.333	1.054	2.214	0.027	0.161
Lawn/Toy	2.667	1.054	2.530	0.011	0.068
Post/Pre	1.333	1.054	1.265	0.206	1.000
Post/Toy	1.667	1.054	1.581	0.114	0.683
Pre/Toy	−0.333	1.054	−0.316	0.752	1.000

**Table 4:** Pairwise comparison of Friedman test ran with animals from high-stereotyping cluster for the frequency of rest/relaxation behaviours in the four experimental treatments. Adjusted significance (Adj. Sig.): significance after Bonferroni correction was applied.

Sample 1/ Sample 2	Test Statistic	Std. Error	Std. Test Statistic	Sig.	Adj. Sig.
Lawn/Post	0.333	1.054	0.316	0.752	1.000
Lawn/Pre	−1.667	1.054	−1.581	0.114	0.683
Lawn/Toy	2.667	1.054	2.530	0.011	0.068
Post/Pre	−1.333	1.054	−1.265	0.206	1.000
Post/Toy	−2.333	1.054	−2.214	0.027	0.161
Pre/Toy	1.000	1.054	0.949	0.343	1.000

## Data Availability

The data presented in this study are available in the present article and supplementary material and are shared with consent and in accordance with all authors.

## Supplementary Materials

The following are available online at https://www.mdpi.com/article/10.3390/ani11082264/s1, Figure S1: FCM concentrations through experimental periods by individual.
