# Supplementary material for: Give Them a Toy or Increase Time out of Kennel at Lawn Areas: What Is the Influence of These Interventions on Police Dogs’ Welfare?
Source: Animals (Basel). 2021 Jul 30;11(8):2264. doi: 10.3390/ani11082264 (PMC8388378; doi:10.3390/ani11082264)
Supplement: Supplementary file 1 [file animals-11-02264-s001.zip › animals-1311716-supplementary.pdf]

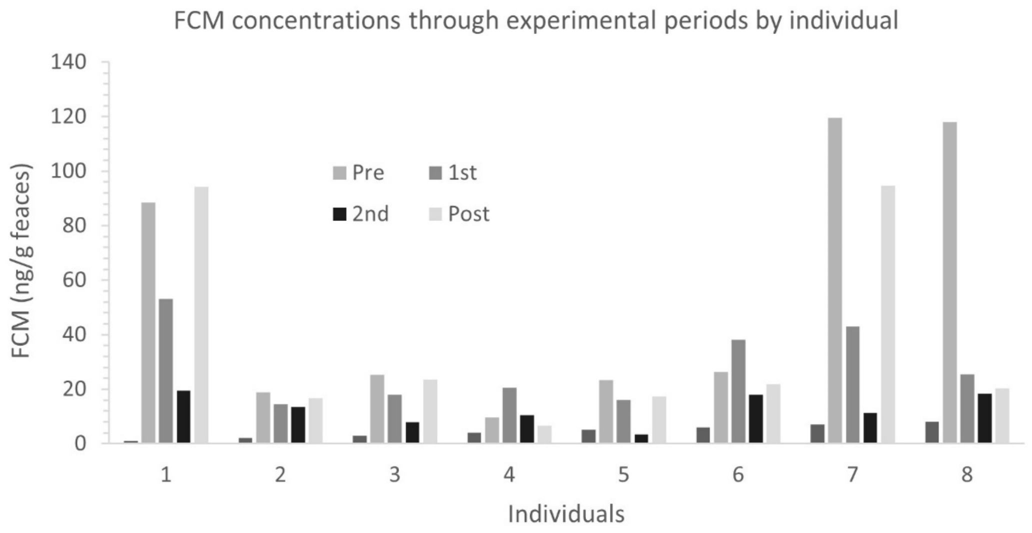

Figure S1. Graph presenting the concentrations of FCM by individual (numbers indicated on Table 1) in our four experimental periods (Pre: pre-interventions; 1st: first intervention; 2nd: second intervention; Post: post-interventions).
